# Exploring Epidemiological Characteristics of Domestic Imported Dengue Fever in Mainland China, 2014–2018

**DOI:** 10.3390/ijerph16203901

**Published:** 2019-10-15

**Authors:** Yujuan Yue, Qiyong Liu

**Affiliations:** State Key Laboratory of Infectious Disease Prevention and Control, National Institute for Communicable Disease Control and Prevention, Chinese Center for Disease Control and Prevention, Beijing 102206, China; yueyujuan@icdc.cn

**Keywords:** epidemiological characteristics, domestic imported dengue fever, time, spatial, crowd

## Abstract

Epidemiological characteristics of domestic imported dengue fever in mainland China, 2014–2018, including time-series, spatial mobility and crowd features, were analyzed. There existed seasonal characteristics from August to November. The 872 domestic imported cases from 8 provinces, located in the southeastern, southwestern and southern coastal or border areas, were imported to 267 counties in 20 provinces of mainland China, located in the outer areas along the southwest-northeast line. The 628 domestic imported cases were still imported to the adjacent counties in the provinces themselves, 234 domestic imported cases were imported to 12 other provinces except the 8 original exported provinces, 493 cases in 2014 reached the peak, and 816 domestic imported cases were from Guangdong (675) and Yunnan (141). Domestic imported cases from Guangdong were imported to 218 counties, and 475 cases from Guangdong were imported to the adjacent counties in Guangdong itself. There were more male cases than female cases except in 2016. Domestic imported cases were clustered from 21 to 50 years old. The top three cases were from farmer, worker and housework or unemployed. The findings are helpful to formulate targeted, strategic plans and implement effective public health prevention and control measures.

## 1. Introduction

Dengue fever, one of the most prevalent mosquito-borne diseases in humans, is mainly transmitted by *Aedes aegyptiand* and *Aedes albopictus* [1]. There are four distinct serotypes for dengue virus, namely DENV 1, 2, 3 and 4 [2]. Dengue fever is endemic in more than 100 countries of Southeast Asia, Africa, Americas, Western Pacific and Eastern Mediterranean regions [3]. Dengue fever has evolved from a sporadic disease to a major public health problem as increasing geographical extension, numbers of cases, and disease severity [3]. It is estimated that 390 million people had dengue virus infections with 96 million cases annually worldwide [1]. 

A total of 655,324 cases and 610 deaths were reported in mainland China from 1978 to 2008. A total of 52,749 cases and six deaths were notified from 2009 to 2014 [4]. A dengue fever outbreak occurred in China, 2014, with 47,127 dengue cases [5]. Dengue fever has spread from the southern coastal areas of Guangdong and Hainan to the relatively northern and western areas including Fujian, Zhejiang, and Yunnan, with shorter outbreak intervals as compared to those before the 1990s [6]. The affected regions expanded gradually over the 10-year period, from the coastal provinces of southern China adjacent to Southeastern Asian countries to the central provinces of China (Henan) [7].

Many studies have explored the characteristics of dengue fever, including crowd features, geographical distribution, temporal distribution, spatio-temporal characteristics and seasonal distribution [1,4,5,8,9,10,11,12,13,14,15,16,17,18,19,20]. Most of the researches focused on indigenous dengue fever, and some researches focused on overseas imported dengue fever [21]. However, there was an almost total lack of epidemiological analyses about domestic imported dengue fever. There was no clear reports about domestic imported dengue fever in mainland China before 2014, so it was a new phenomenon of dengue fever in mainland China after 2014 with the aggravation of dengue fever in China and the maturity of dengue fever surveillance technology. With the severe prevalence and spatial diffusion of dengue fever in China in recent years, it is significant to investigate epidemiological dynamics of domestic imported dengue fever. A better understanding of domestic imported dengue fever can help mastering the prevalence and mobility of dengue fever and planning resource allocation for dengue fever prevention and control. Therefore, this study analyzed epidemiological characteristics of domestic imported dengue fever in mainland China, 2014–2018, including time-series features, spatial mobility features and crowd features. 

## 2. Materials and Methods

### 2.1. Data Collection

Dengue cases were defined based on clinical diagnosis and laboratory confirmation according to diagnostic criteria and principle of management for dengue (WS 216-2001, before 2008. “WS 216-2001” is a number of public health standard in the People’s Republic of China. “WS”, the capital words of Wei Sheng, stands the meaning of public health in Chinese) or diagnostic criteria for dengue (WS 216-2008, after 2008) [22,23].

Dengue fever is a vector-borne notifiable disease. Dengue cases are reported to Chinese Center for Disease Control and Prevention (China CDC) by law through Chinese National Notifiable Infectious Disease Reporting Information System (CNNDS). Dengue case report includes age, sex, occupation, national code of current address, date of illness onset, remarks, etc. There are several kinds of occupations as farmer, businessman, housework or unemployed, etc. Daily dengue case reports from 1 January 2014 to 13 December 2018 were obtained from CNNDS. The vector data of Chinese administrative divisions, which were used for geographical mapping, were provided by CNNDS.

Ethics Statement: No human or animal samples were included in the research presented in this article; therefore ethical approval was not necessary for this research.

### 2.2. Data Processing

According to the remarks of dengue case reports, dengue cases were divided into indigenous dengue cases, overseas imported dengue cases, domestic imported dengue cases and other dengue cases. Indigenous dengue cases refer to the cases who did not leave the local counties (the current addresses) within 14 days before illness onset. Overseas imported dengue cases refer to the cases who went to foreign countries or regions within 14 days before illness onset where dengue fever was prevalent. Domestic imported dengue cases refer to the cases who left the local counties (the current addresses) and went to other domestic counties within 14 days before illness onset where dengue fever was prevalent. Other dengue cases refer to the cases that we are not sure how to classify. Domestic imported dengue cases were studied in this research. Finally, domestic imported dengue cases were among June to December. The case remark showed the exported province or city or county, which could be geocoded and matched to administrative boundaries (province or city or county) for spatial analysis using ArcGIS version 10.5 (ESRI, Redlands, CA, USA) [24]. The national code of current address showed the imported county, which could be matched to the county-level administrative boundaries for spatial analysis using ArcGIS version 10.5 [24]. 

### 2.3. Data Analysis

Time-series analyses and crowd analyses for domestic imported dengue cases were conducted using IBM SPSS Statistics version 24.0 (IBM Corp., Armonk, NY, USA). Spatial mobility analyses for domestic imported dengue cases were conducted using ArcGIS version 10.5 [24]. 

## 3. Results

### 3.1. Time-Series Analyses

**Figure 1:**
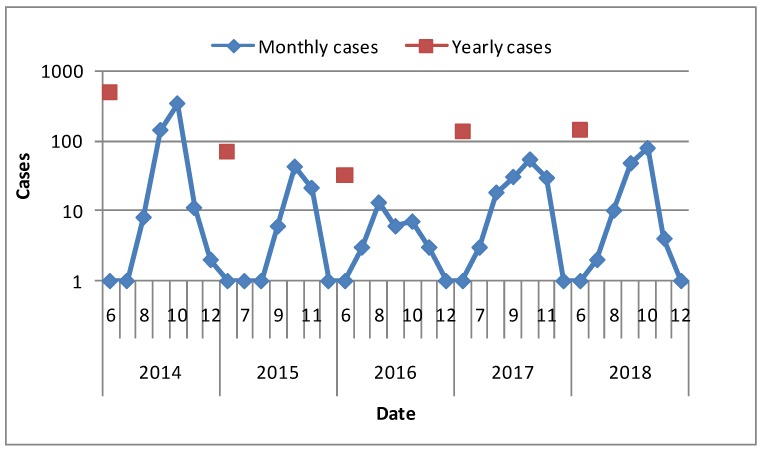
Time-series mapping of domestic imported dengue cases (domestic imported dengue cases were among June to December during 2014–2018).

There existed seasonal characteristics for domestic imported dengue cases from August to November. Over the past five years, the numbers of cases had declined and increased again. The largest number of cases, 493 cases, occurred in 2014, accounting for 56.5% of the total (Figure 1).

### 3.2. Spatial Mobility Analyses

**Figure 2:**
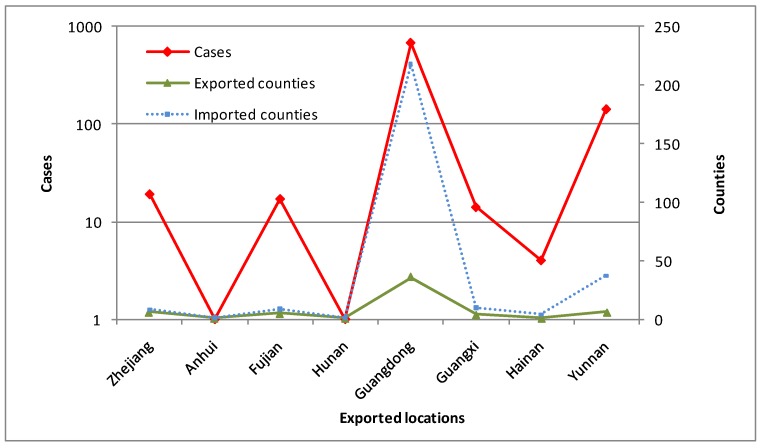
Cases and counties in central exported locations.

**Figure 3:**
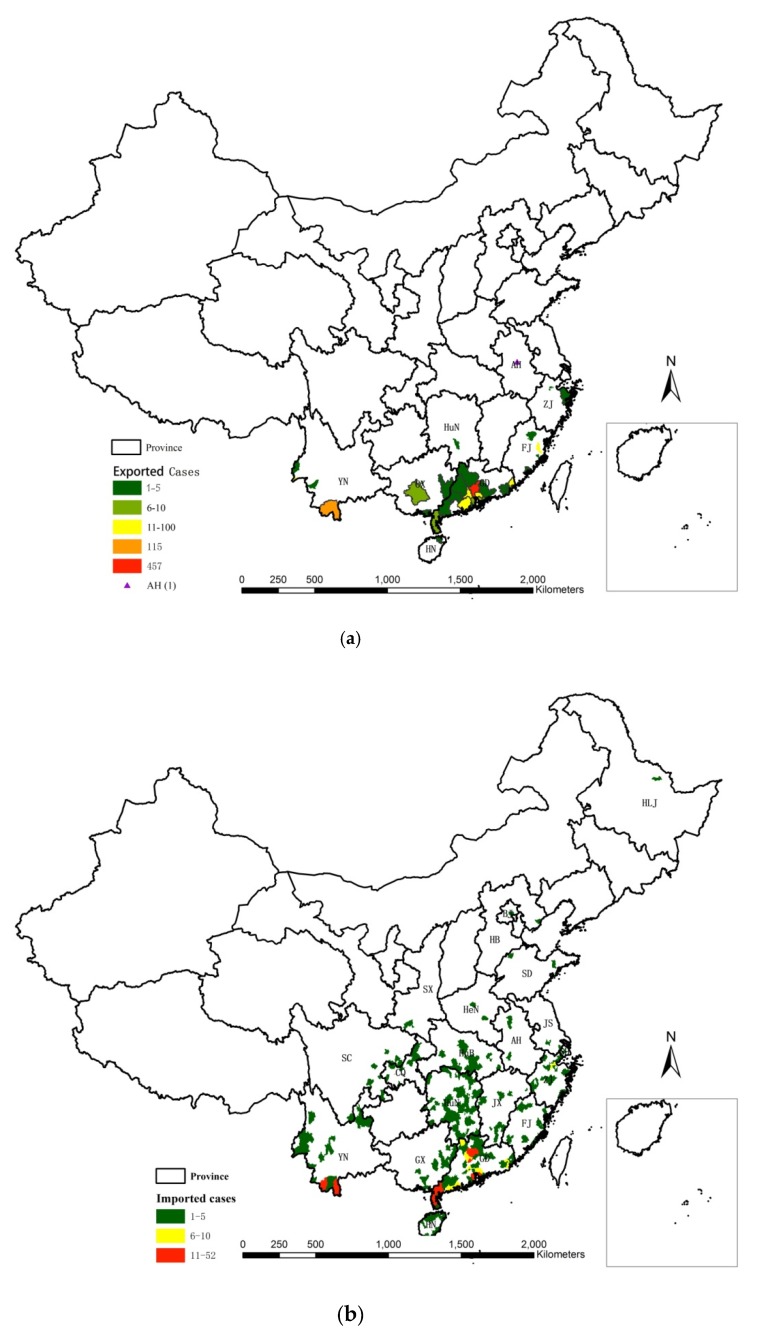

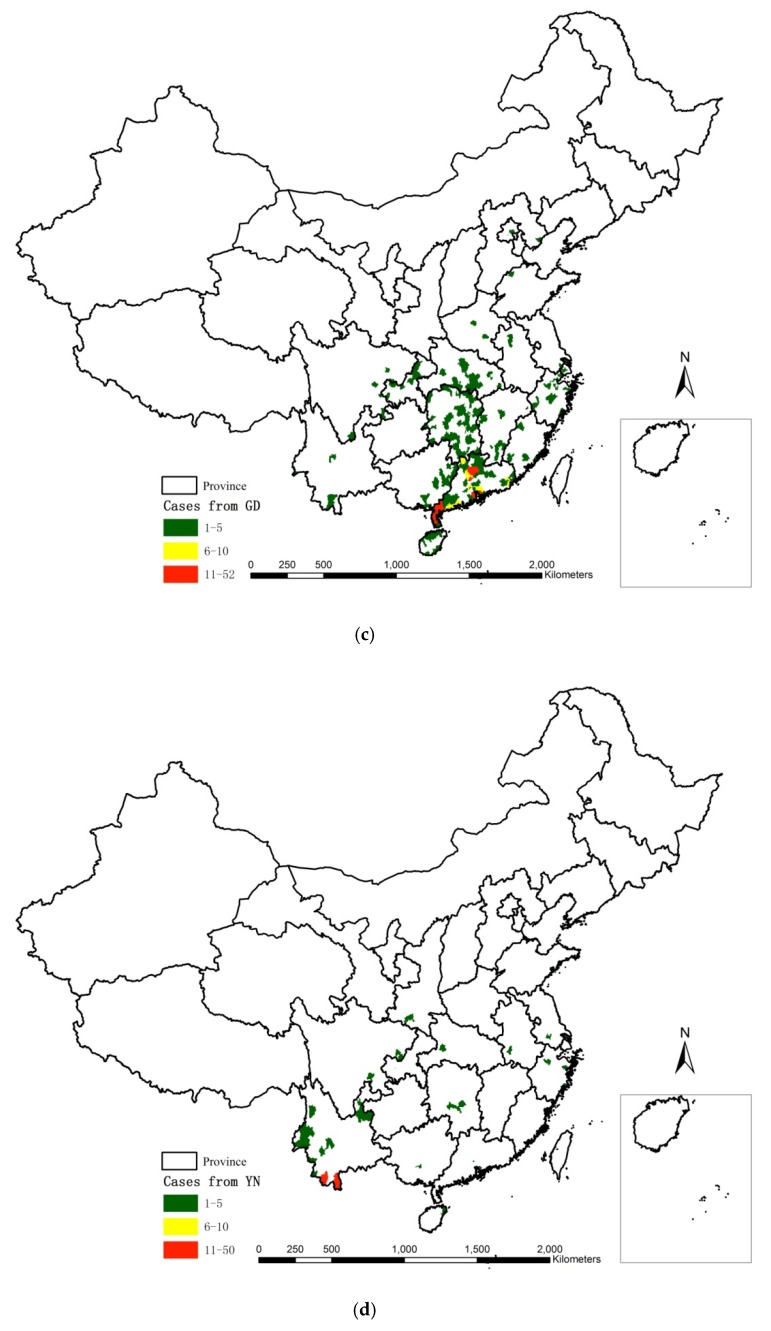
Spatial mapping of domestic imported dengue cases. (**a**) Spatial distribution of exported cases. (**b**) Spatial distribution of imported cases. (**c**) Cases from GD. (**d**) Cases from YN. (Note: YN: Yunnan; GD: Guangdong; GX: Guangxi; HN: Hainan; FJ: Fujian; SC: Sichuan; CQ: Chongqing; HuN: Hunan; HuB: Hubei; JX: Jiangxi; ZJ: Zhejiang; AH: Aihui; JS: Jiangsu; SH: Shanghai; HeN: Henan; HB: Hebei; SD: Shandong; SX: Shanxi; BJ: Beijing; HLJ: Heilongjiang).

There were 872 domestic imported dengue cases in China, 2014–2018, which were from 8 provinces, located in the southeastern, southwestern and southern coastal or border areas in mainland China (Figure 2 and Table 1). Domestic imported cases and counties were mainly from Guangdong (675) and Yunnan (141). Some 457 cases were from the total counties in Guangzhou City, Guangdong Province and 101 cases were from Jinghong City, Xishuangbanna Dai Autonomous Prefecture, Yunnan Province (Table 1 and Figure 3a).

These 872 cases were imported to 267 counties in 20 provinces, located in the outer areas along the southwest-northeast line of mainland China. Domestic imported dengue cases were imported to 59 counties (22.1% of the total imported counties) in the other 12 provinces except the 8 original exported provinces. Also, 675 cases from 36 counties in Guangdong were imported to 218 counties in 18 provinces in northern, central and southern areas of mainland China. Most of them, 475 cases, were imported to 71 adjacent counties in Guangdong itself, followed by 68 cases to 43 counties in Hunan, 20 cases to 15 counties in Hubei and 20 cases to 17 counties in Zhejiang, respectively (Figure 3c). The 141 cases from 6 counties in Yunnan were imported to 37 counties in 13 provinces in central and southern areas of China. Most of them, 118 cases, were imported to 16 adjacent counties in Yunnan itself (Figure 3d). The 19 cases from Zhejiang were imported to 8 counties in Zhejiang and the adjacent provinces of Jiangxi and Fujian. Most of them, 17 cases, were imported to 6 adjacent counties in Zhejiang itself. The 17 cases from Fujian were imported to 9 counties in Fujian, Heilongjiang and Shandong. Most of them, 15 cases, were imported to 7 adjacent counties in Fujian itself. The 14 cases from Guangxi were imported to 10 counties in Guangxi, Guangdong and Zhejiang. Most of them, 9 cases, were imported to 5 counties in Guangdong. 4 cases from Hainan were imported to 4 counties in Fujian, Hunan and Guangdong. One case from Anhui were imported to 1 county in Guangdong. One case from Hunan was imported to the adjacent county in Hunan itself. 

### 3.3. Crowd Analyses

By gender, there are 522 male cases and 350 female cases. However, there were more female cases than male cases in 2016. By age group, domestic imported dengue cases were clustered in the 21–30, 31–40 and 41–50 age groups, with the ratio of 31.0%, 22.5%, 18.2%, respectively. By career, the top three cases were from farmer, worker and housework or unemployed, with the ratio of 24.4%, 13.4%, 13.4%, respectively (Table 2, Table 3 and Table 4).

## 4. Discussion

**Figure 4:**
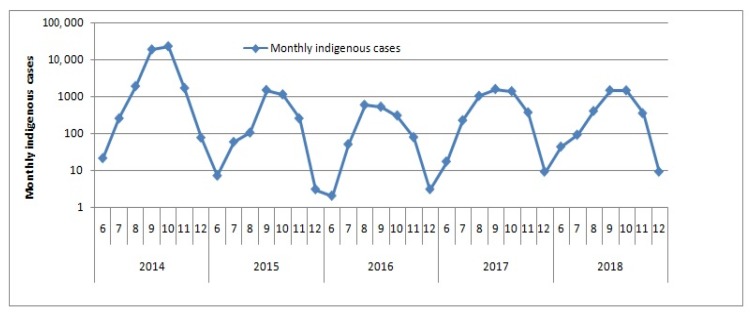
Time-series mapping of monthly indigenous dengue cases.

There were 872 domestic imported dengue cases in mainland China during 2014–2018, which were analyzed in this research. The total of domestic imported dengue cases were among June to December in this research. Temporal distribution and seasonal characteristics from August to November of domestic imported dengue fever were similar with that of indigenous dengue fever (Figure 4) [5,7]. Indigenous dengue cases and domestic imported dengue cases had both declined and increased again in the study period. Therefore, the more indigenous dengue cases, the more domestic imported dengue cases. A dengue fever outbreak occurred in Guangdong, 2014 [5,18]. Indigenous dengue cases (77.9%) and domestic imported dengue cases (56.5%) in 2014 both reached peaks during 2014–2018 [21]. There are several complex factors which may have contributed to dengue fever outbreaks. With the development of economy and society, there are more and more people migrating between China and dengue endemic countries such as Philippines, Burma, Thailand, etc. in recent years. Then overseas imported dengue cases may cause more domestic dengue cases. Climate changes have also increased mosquito density and expanded geographic distribution and seasonal distribution of *Aedes spp* mosquitoes. Moreover, more and more attentions have been paid to dengue fever and more sensitive and rapid laboratory tests have been introduced to more counties, which have also contributed to the increase of reported dengue cases. 

There was a big spatial mobility for domestic imported dengue cases, from 8 provinces in the southeastern, southwestern and southern coastal or border areas in mainland China to the other 12 provinces in the outer areas along the southwest-northeast line of mainland China. Indigenous dengue fever was spreading from the southwestern border, southern provinces and the southeastern coastal provinces to the central and northern regions in 2014–2018 [21]. Spatial spreading of dengue cases is closely related to economic development, traffic development, population mobility and climate warming, etc. [13,14,16,17,25,26], which poses a potential serious threat to public health in China.

The 816 domestic imported cases were from Guangdong (77.4%) and Yunnan (16.2%). Domestic imported cases originating from Guangdong were imported to large-scale spatial counties (81.6% of the total imported counties) in northern, central and southern areas of mainland China. Dengue fever epidemic in Guangdong led dengue fever epidemics in mainland China. Most of the dengue cases were located in Guangdong and Yunnan [4]. However, 628 domestic imported cases were still imported to the adjacent counties in the provinces themselves. Also, 234 domestic imported cases were exported from four domestic provinces of mainland China to other provinces: Guangdong (96.2%), Yunnan (2.1%), Guangxi (1.3%) and Hainan (0.4%). Of those interprovincial case movements, most (96.6%) occurred in 2014 [7]. Therefore, we should focus on the prevention, control and monitoring of dengue fever in Guangdong and Yunnan, especially Guangzhou City in Guangdong, as well as Jinghong City in Yunnan. 

There were more male domestic imported cases than female cases except in 2016. There were more male dengue cases than female cases during 2005–2015, except in 2013 and 2014 [18]. There was a strong male predominance among imported cases and an almost equal gender distribution for indigenous cases [7]. Domestic imported cases were clustered in those who were 21 to 50 years old. Most dengue cases occurred in individuals aged between 21 and 50 years old [18]. This might reflect a population of younger working male adults who tend to travel more domestically and regionally and thereby have more exposure risk to dengue fever [7]. The top three cases were from farmer, worker and housework or unemployed. Dengue cases were involved in housework or unemployed, businessmen, retired, workers, farmer, etc. [18]. Dengue fever is a systemic viral infection transmitted by mosquitoes of the *Aedes* genus [27]. Dengue fever is related with mosquito density directly [2,13,28]. Dengue fever is also closely related with environmental and socio-economic conditions, such as land use and land cover type, sanitation status, population density, ventilation conditions, etc. [5]. Water and a suitable temperature are essential factors for the larvae of dengue virus vector of *Aedes* mosquito. Human activities are closely related to house address, work units and other places of activity. Farmers generally work in vegetable fields, farmlands and arable lands, which are favorable for mosquito vector breeding, thus farmers have high bite rates. Workers work in relatively complex and poor environments, and also have comparatively high bite rates. Housework or unemployed may spend most of their time at home. In order to make rational and effective use of resources, investments in mosquito control may be less in personal houses than in public workplaces, and mosquito bites are more possible. Moreover, housework or unemployed may lack sufficient knowledge of dengue fever and mosquito vectors. In order to cope with dengue fever in China, it is necessary to strengthen knowledge of dengue prevention and control among these occupations. 

## 5. Conclusions

Epidemiological characteristics of domestic imported dengue fever in China, 2014–2018, were analyzed in this study. The 872 domestic imported cases from 8 provinces were imported to 267 counties in 20 provinces. Also, 816 domestic imported cases were from Guangdong (77.4%) and Yunnan (16.2%). There were more male cases than female cases. Most dengue cases occurred in those aged between 21 and 50 years old. The top three careers were farmer, worker and housework or unemployed. Grasping epidemiological characteristics of domestic imported dengue fever is helpful to formulate targeted, strategic plans to implement effective public health prevention and control measures. 

## Figures and Tables

**Table 1:** Exported locations to imported locations.

Exported Location	Cases	Numbers of Exported Counties	Numbers of Imported Provinces	Numbers of Imported Counties
Zhejiang	19	6	3	8
Aihui	1	1	1	1
Fujian	17	5	3	9
Hunan	1	1	1	1
Guangdong	675	36	18	218
Guangxi	14	4	3	10
Hainan	4	1	3	4
Yunnan	141	6	13	37

**Table 2:** Gender distribution of domestic imported dengue fever from 2014–2018.

Year	Male Cases	Female Cases
2014	317	176
2015	41	29
2016	10	22
2017	79	56
2018	75	67

**Table 3:** Age group distribution of domestic imported dengue fever from 2014–2018.

Age Group	Cases in 2014	Cases in 2015	Cases in 2016	Cases in 2017	Cases in 2018
0–10	8	0	1	2	2
11–20	65	11	3	19	17
21–30	164	15	6	39	46
31–40	114	16	1	34	31
41–50	85	15	9	24	26
51–60	38	11	6	13	15
61–70	13	2	5	3	4
71+	6	0	1	1	1

**Table 4:** Career distribution of domestic imported dengue fever from 2014–2018.

Career	Cases in 2014	Cases in 2015	Cases in 2016	Cases in 2017	Cases in 2018
Cadre	16	5	2	12	7
Worker	64	7	5	20	21
Housework or unemployed	79	4	5	12	17
Retiree	7	4	1	5	4
Migrant laborer	37	6	0	5	4
Farmer	115	19	8	32	39
Businessman	47	9	1	21	18
Student	56	7	3	9	10
Medical staff	3	2	2	4	2
Else	24	3	0	5	7
Unavailable	45	4	5	10	13
